# Boosting elemental mercury capture *via* an iodine-mediated pathway over a ternary BiOI-MnO_*x*_-TiO_2_ catalyst

**DOI:** 10.1039/d5ra09048b

**Published:** 2026-02-02

**Authors:** Wenju Li, Dan Peng, Anchao Zhang

**Affiliations:** a School of Energy and Power Engineering, Zhengzhou Electric Power College Zhengzhou 450000 P.R. China; b Henan University Engineering Research Center for Power Energy Conservation and Emission Reduction, Zhengzhou Electric Power College Zhengzhou 450000 P.R. China pengdanhust@163.com; c School of Mechanical and Power Engineering, Henan Polytechnic University Jiaozuo 454003 P.R. China

## Abstract

Elemental mercury (Hg^0^) emission from coal combustion flue gas poses significant environmental and health risks due to its high volatility, persistence, and toxicity. In this study, a novel ternary BiOI-MnO_*x*_-TiO_2_ (BiMnTi) composite catalyst was successfully synthesized *via* a simple three-step method for efficient Hg^0^ removal under dark conditions. The composite catalysts were characterized by SEM-EDS, HRTEM, XRD, H_2_-TPR, N_2_ adsorption–desorption, FTIR, XPS, and EPR. The BiOI-MnO_*x*_-TiO_2_ composite exhibited superior Hg^0^ removal efficiency (>97%) over a wide temperature range of 50–200 °C, and showed excellent resistance to SO_2_ and NO poisoning. Characterization results confirmed that the introduction of BiOI effectively increased the proportion of Mn^4+^ content and surface chemisorbed oxygen (O_β_) and promoted the formation of oxygen vacancies. XPS and H_2_-TPR analyses further demonstrated enhanced electron transfer between BiOI and MnO_*x*_-TiO_2_, as well as improved redox properties. Mechanistic studies revealed that the synergistic interaction between BiOI and MnO_*x*_-TiO_2_ facilitated electron transfer at the interface, promoting the oxidation of I^−^ to active iodine species, which subsequently reacted with adsorbed Hg^0^ to form stable HgI_2_. This work provides a promising strategy for designing efficient and sulfur-resistant catalysts for Hg^0^ removal in non-photocatalytic environments.

## Abstract 2

A ternary BiOI-MnO_*x*_-TiO_2_ catalyst enables efficient capture of Hg^0^*via* an iodine-mediated pathway, achieving 97% of Hg^0^ removal efficiency over 50–200 °C with strong SO_2_ and NO resistance.

## Graphical abstract

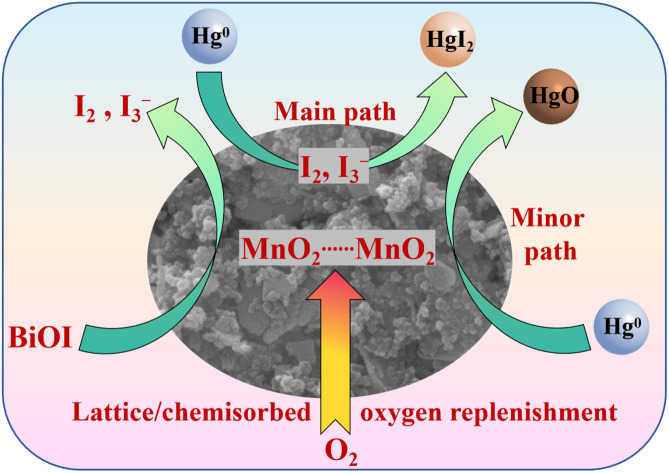

## 1. Introduction

In recent years, the effective removal and transformation of persistent environmental pollutants have attracted widespread attention due to the significant risks they pose to ecology and human health.^1–3^ Among these, mercury has an atmospheric residence time ranging from several months to years, leading to long-term risks to ecosystems and human health. This persistent hazard is tragically illustrated by historical public health incidents such as Minamata disease. Among anthropogenic sources, mercury emissions from coal combustion flue gas represent a major environmental challenge and constitute the largest source of global anthropogenic mercury release.^4^ In flue gas, mercury exists primarily in three forms: elemental mercury (Hg^0^), oxidized mercury (Hg^2+^), and particulate-bound mercury (Hg^p^).^5^ While Hg^2+^ can be effectively captured by wet flue gas desulfurization (WFGD) systems and Hg^p^ is removable *via* electrostatic precipitators or fabric filters,^6^ the efficient removal of Hg^0^ remains particularly difficult due to its high volatility and low solubility in water. Therefore, developing efficient and economical control technologies for mercury emissions is of significant importance.

Activated carbon (AC) injection has been widely investigated and implemented as a potential method for mercury removal.^7,8^ However, its high operational cost and adverse impact on fly ash quality and downstream equipment have motivated the search for alternative, cost-effective materials. In recent years, numerous metal oxide-based catalysts and sorbents, such as MnO_*x*_/Alumina,^6^ Ce-MnO_*x*_/TiO_2_,^9^ CeO_2_-MnO_*x*_^10^ and Mn_2_Fe_1_Ce_*x*_/C,^11^ have shown promising performance in Hg^0^ removal. Among these, manganese-based oxides (MnO_*x*_) are considered highly attractive due to their multi-valency, high adsorption capacity, and strong oxidation activity at low temperatures.^12^ Nevertheless, the susceptibility of MnO_*x*_ to SO_2_ poisoning remains a major obstacle to its practical application.

To enhance the sulfur resistance of manganese-based catalysts, the introduction of secondary metal components (*e.g.*, Mo,^13^ Cu,^14^ Ce,^15^ Sn,^16^ and Ru^17^) has been widely attempted. For instance, Yang *et al*. reported that Mn–Fe_2_O_3_ spinel exhibited not only high Hg^0^ capture efficiency but also significantly improved SO_2_ tolerance compared to pure MnO_*x*_.^18^ Xu *et al*. demonstrated that the addition of Fe to Sn-MnO_*x*_ greatly enhanced both Hg^0^ removal performance and sulfur resistance.^19^ Chen *et al*. also found that Cr-modified MnO_*x*_-TiO_2_ achieved high mercury removal efficiency and SO_2_ durability at low temperatures.^20^ These studies confirm that rational design for sorbents is an effective strategy for developing high-performance mercury removal materials.

Recently, bismuth oxyhalides (BiOX, X = Cl, Br, I) have attracted increasing attention as a family of layered semiconductors with exceptional visible-light photocatalytic activity, chemical stability, and tunable electronic properties.^21,22^ In particular, BiOI has been extensively studied for environmental remediation and photocatalytic applications. Guan *et al*. constructed a flower-like BiOI/Bi_5_O_7_I heterojunction that exhibited superior photocatalytic mercury removal performance under visible light.^23,24^ Our previous studies also indicated that the Hg^0^ removal activity followed the order BiOI > BiOBr > BiOCl, and that further modification with Ag significantly enhanced the photocatalytic oxidation activity of BiOI/ZnFe_2_O_4_.^25,26^ Most previous studies on BiOI-based materials for Hg^0^ removal have focused on photocatalytic systems, in which light irradiation is essential for activating iodine species and surface redox reactions.^27–29^ However, the potential role of BiOI in non-photocatalytic Hg^0^ removal, especially under dark conditions and in combination with transition metal oxides, has rarely been explored. This work therefore investigates the Hg^0^ removal behavior of a BiOI-MnO_*x*_-TiO_2_ composite in the absence of light, aiming to clarify whether iodine chemistry can be activated through interfacial interactions rather than photoexcitation.

Inspired by above considerations, this study aims to develop a novel BiOI-modified MnO_*x*_-TiO_2_ composite *via* a simple wet grinding method for enhanced Hg^0^ removal in dark conditions. The MnO_*x*_-TiO_2_ and BiOI components were first prepared separately by deposition–precipitation and coprecipitation methods, respectively, and then composited through mechanical grinding. The obtained materials were systematically characterized, and their Hg^0^ removal performance was evaluated under a simulated flue gas atmosphere. The impact of component adding ratio, catalyst dose, reactive temperature, and flue gas composition on Hg^0^ removal efficiency was thoroughly investigated. Finally, the Hg^0^ removal mechanism was proposed through the results of experiments.

## 2. Experimental section

### 2.1. Synthesis of materials

#### 2.1.1. Preparation of BiOI catalyst

BiOI catalyst was prepared *via* a typical coprecipitation method. Specifically, 13.78 g of Bi(NO_3_)_2_·5H_2_O was dissolved in a mixed solution of 600 mL deionized water and 200 mL ethylene glycol. After ultrasonic treatment for 30 min, 50 mL of KI solution (4.72 g) was added dropwise to the Bi(NO_3_)_2_ solution under continuous stirring. The mixture was stirred for 90 min and then allowed to stand for 12 h. The resulting precipitate was filtered and repeatedly washed with ethanol and deionized water until neutral pH was achieved. Finally, the product was dried in an oven at 75 °C for 24 h.

#### 2.1.2. Preparation of MnO_*x*_-TiO_2_ catalyst

MnO_*x*_-TiO_2_ catalyst was synthesized using a deposition–precipitation method, with the mass ratio of MnO_*x*_ to TiO_2_ fixed at 20%. First, 10 g of commercial P25 TiO_2_ was dispersed in 120 mL deionized water and stirred for 30 min. Then, 2.91 mL of Mn(NO_3_)_2_·4H_2_O was added dropwise to the TiO_2_ suspension under vigorous stirring. Subsequently, a mixture of NH_3_·H_2_O (15 wt%) and NH_4_HCO_3_ (15 wt%) was added to adjust the pH to 10.0. The suspension was aged at 60 °C for 2 h, then filtered and washed to neutrality. The solid was dried at 110 °C for 24 h and finally calcined at 450 °C for 5 h in air. The resulting catalyst was denoted as MnTi.

#### 2.1.3. Fabrication of ternary BiOI-MnO_*x*_-TiO_2_ catalyst

The wet milling strategy was deliberately selected to construct an intimate and strongly interacting interface between BiOI and MnO_*x*_-TiO_2_ under light-free conditions. Compared with hydrothermal, solvothermal, or impregnation methods, mechanical milling in an ethanol medium enforces close microscale or even nanoscale contact between the pre-formed components, which would be beneficial for interfacial electron transfer. The ternary BiOI-MnO_*x*_-TiO_2_ catalyst was fabricated *via* a wet grinding method. Different mass ratios of BiOI to MnO_*x*_-TiO_2_ (1 : 9, 2 : 8, 3 : 7, 4 : 6, 5 : 5) were achieved by varying the quantities of each component. Briefly, appropriate amounts of BiOI, MnO_*x*_-TiO_2_, and absolute ethanol were mixed and thoroughly ground in an agate mortar for over 30 min. The mixture was then dried in a porcelain boat at 70 °C for 4 h. The resulting BiOI-MnO_*x*_-TiO_2_ sample was ground and sieved to 100 mesh. The samples were labeled as BiMnTi-1, BiMnTi-2, BiMnTi-3, BiMnTi-4, and BiMnTi-5, respectively. For comparison, pure BiOI and MnTi were also ground separately using the above procedure and were designated as BiOI-g and MnTi-g, respectively, to evaluate the effect of grinding on the sample structure.

### 2.2. Evaluation of catalyst activity

The experimental equipment consisted of a simulated flue gas system, a mercury source, a fixed-bed reactor, a Hg^0^ concentration analysis system and a tail gas treatment device. The detailed information about experimental setup and process can be referred to the previous work.^30^ For the evaluation of catalyst activity, a glass reactor with an inner diameter of about 4 mm and a length of 700 mm was employed. The simulated flue gases containing N_2_, O_2_, CO_2_, SO_2_ (when used), and NO (when used) were supplied from compressed gas cylinders and precisely controlled by mass flow controllers (MFCs). In the experiment, the basic components of the flue gas were balance N_2_, 12% of CO_2_ and 6% of O_2_, and 72 ± 3 µg m^−3^ Hg^0^ at standard atmospheric pressure. The total flow rate was maintained at 1.5 L min^−1^. The Hg^0^ concentration was recorded by a VM-3000 mercury analyzer (German Mercury Instrument Co., Ltd) and the Hg^0^ removal efficiency (*η*, %) was calculated as follows:

1 *η* = (1 − *C*_out_/*C*_in_) × 100%

where *C*_in_ and *C*_out_ represent the concentration of Hg^0^ (µg m^−3^) at the inlet and outlet of the fixed-bed reactor, respectively.

### 2.3. Samples characterization

Scanning electron microscopy (SEM, Quanta 250) coupled with an energy dispersive X-ray spectrometer (EDS) was employed to characterize the morphology and elemental distribution. The microtopography were obtained using a JEOL 2100 Transmission Electron Microscope. X-ray powder diffraction (XRD) patterns were recorded on a D8 Advance diffractometer with a scanning range of 5° to 90°. H_2_ temperature-programmed reduction (H_2_-TPR) was conducted on an AutoChem II 2920 apparatus. Prior to analysis, samples were pretreated at 200 °C for 2 h under an Ar atmosphere. The reduction was carried out using a gas mixture of 15% H_2_ and 85% Ar (30 mL min^−1^) while heating from 50 to 800 °C at a rate of 10 °C min^−1^. The specific surface area, pore volume, and pore size distribution were determined by N_2_ adsorption–desorption using a Quantachrome instrument (USA). Fourier transform infrared (FTIR) spectra were acquired on a Nicolet 5700 spectrometer (USA) in the range of 4000−400 cm^−1^. X-ray photoelectron spectroscopy (XPS) was performed using an Escalab 250xi spectrometer, with the C 1 s peak at 284.6 eV used as an internal reference for calibration. Electron paramagnetic resonance (EPR, JES FA200) tests were performed to confirm the presence of oxygen vacancy.

## 3. Results and discussion

### 3.1. Hg^0^ removal performance of catalysts

**Fig. 1:**
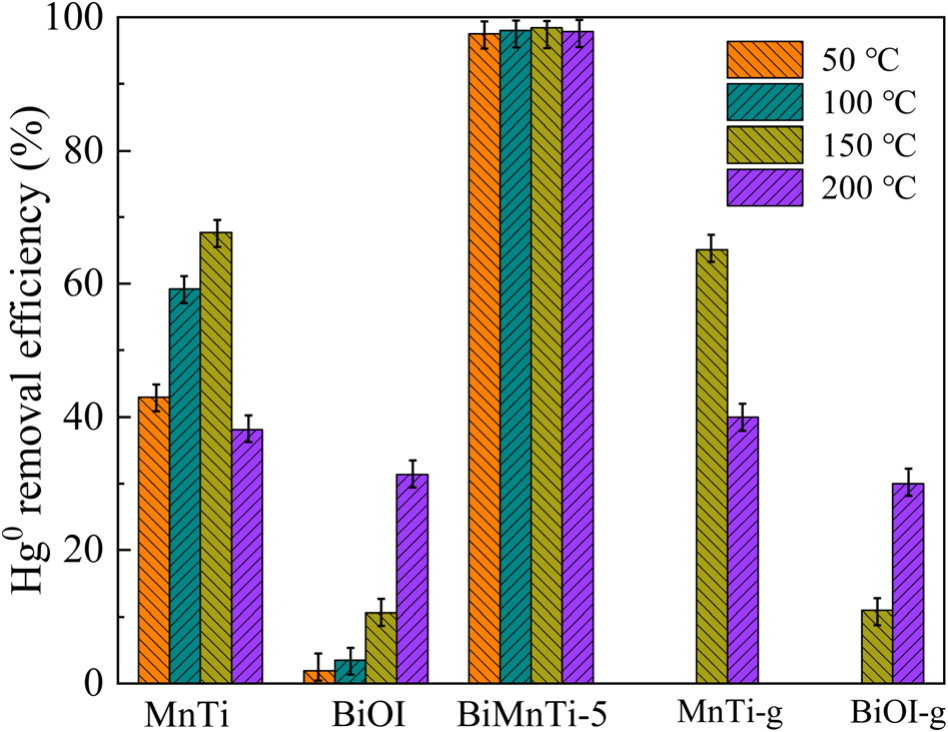
Hg^0^ removal efficiency of catalysts at different reaction temperatures.

The effect of reaction temperature on Hg^0^ removal efficiency is shown in Fig. 1. To obtain the accurate Hg^0^ removal efficiency, the reaction time was set at 1 h and the catalyst mass was 0.1 g. MnTi achieved a removal efficiency of 67.7% at 150 °C. However, its efficiency decreased when the temperature was raised to 200 °C, likely due to the combined effects of inhibited Hg^0^ adsorption and desorption of formed HgO from the catalyst surface.^30^ Although MnTi contained high-valence Mn^4+^ and lattice oxygen in its bulk phase, these active species were not readily accessible for reaction with highly stable Hg^0^ atoms. The oxidation of Hg^0^ required breaking its chemical inertness, a process associated with a high energy barrier on the pure MnO_2_ surface, resulting in a slow reaction rate. The Hg^0^ removal efficiency of BiOI increased with reaction temperature and reached only 31.4% at the highest temperature tested. In the absence of light, BiOI itself lacks strong oxidizing power and cannot oxidize Hg^0^ by providing lattice oxygen or through valence change, unlike MnO_2_. Its layered structure may offer a certain capacity for the physical adsorption of Hg^0^. However, this capacity was limited, and the adsorption was weak and reversible, leading to easy desorption. By comparison, the modification of MnTi with BiOI (BiMnTi-5) resulted in a significant enhancement on Hg^0^ removal.^31^ The BiMnTi-5 composites maintained above 97% of Hg^0^ removal efficiencies across a broad temperature range from 50 to 200 °C. Moreover, it was observed that the grinding of MnTi and BiOI alone did not improve Hg^0^ removal efficiency, implying that the enhanced activity originated from synergistic interactions between the two components. Thus, the above results indicated that the addition of BiOI significantly enhanced the catalytic activity of BiMnTi catalysts. Since the materials used for mercury removal are usually in the dust removal process (100–200 °C), the subsequent related experiments were conducted at 150 °C.

**Fig. 2:**
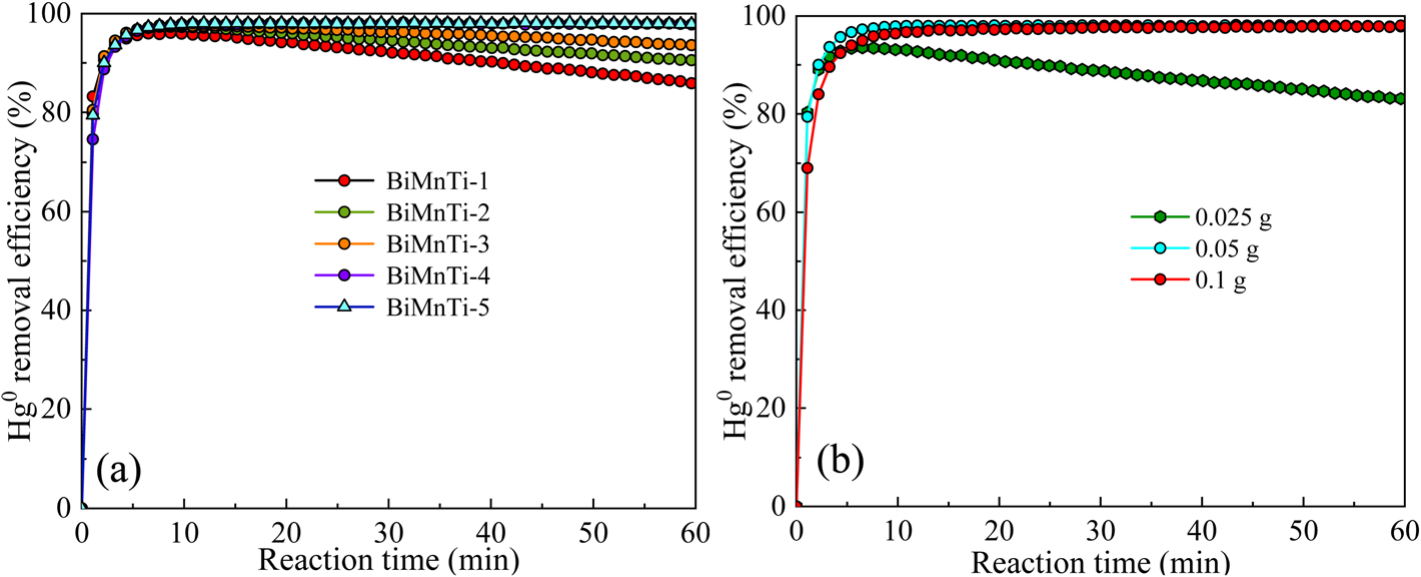
Effects of BiOI/MnO*x-*TiO_2_ mass ratio (a) and catalyst dosage (b) on Hg^0^ removal efficiency.

Fig. 2b illustrates the effect of BiOI/MnO_*x*_*-*TiO_2_ mass ratio on Hg^0^ removal efficiency. It was found that Hg^0^ removal efficiency increased gradually with BiOI content increasing, indicating the important role of BiOI in Hg^0^ removal. The optimal performance (98%) was achieved with a BiOI to MnO*_x_-*TiO_2_ mass ratio of 1 : 1 (BiMnTi-5). Fig. 2b shows the effect of catalyst dosage on Hg^0^ removal efficiency. 0.025 g of BiMnTi-5 resulted in a poor performance of Hg^0^ removal due to limited active sites available for mercury capture. As the adsorbent dosage increased to 0.05 g and 0.1 g, the removal efficiency rose significantly, as more active sites become accessible for mercury adsorption. This demonstrated that adsorbent dosage significantly influences Hg^0^ removal efficiency and highlighted the importance of optimizing adsorbent dosage to achieve cost-effective and efficient Hg^0^ control in industrial applications.

**Fig. 3:**
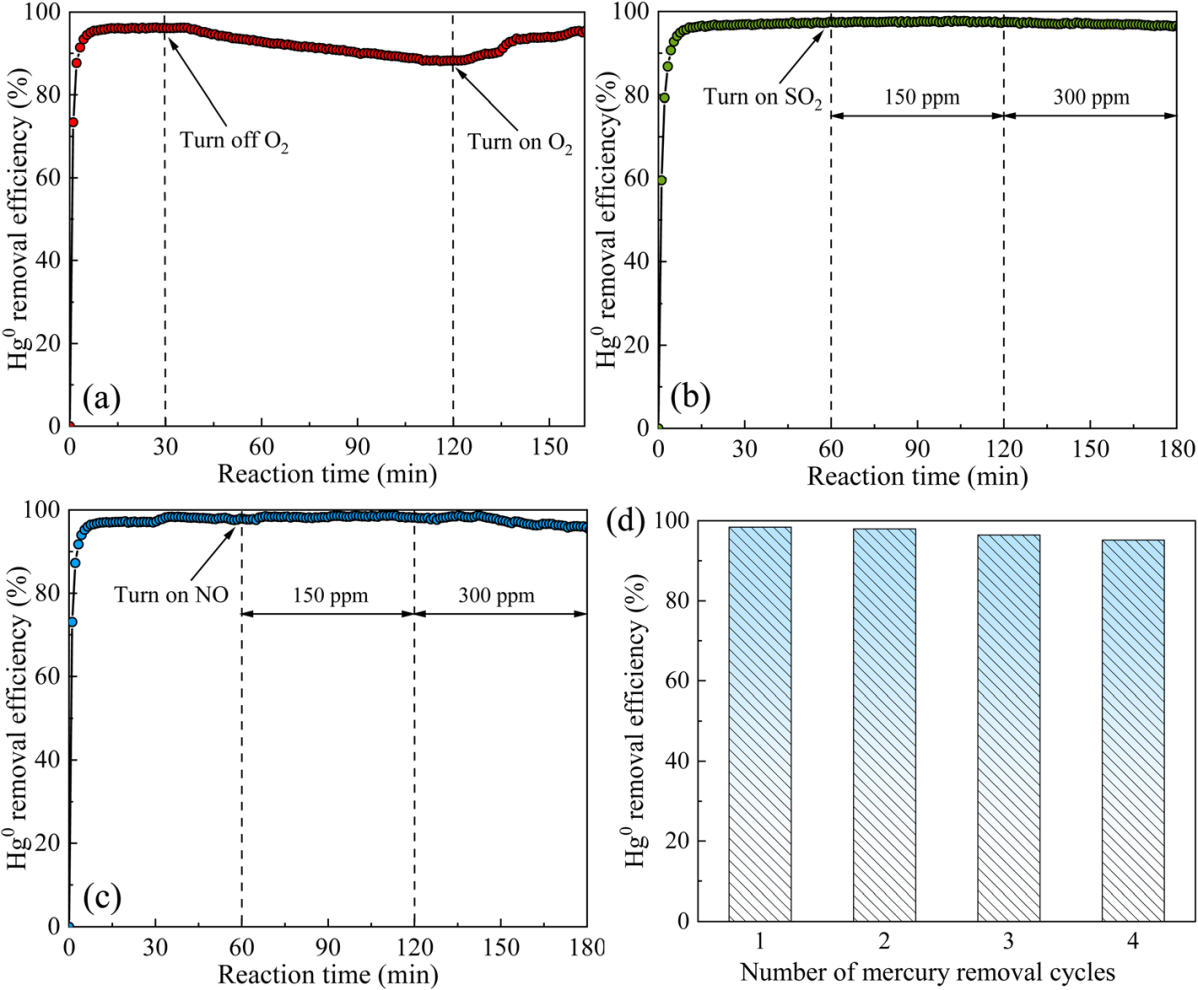
Effects of O_2_ (a), SO_2_ (b) and NO (c) on Hg^0^ removal efficiency; the Hg^0^ removal efficiency for four cycles (d).

The influences of flue gas components (O_2_, SO_2_ and NO) on Hg^0^ removal over BiMnTi-5 are presented in Fig. 3. As shown in Fig. 3a, under baseline conditions, the Hg^0^ removal efficiency was approximately 98% within the first 30 min. While in the absence of O_2_, the efficiency gradually decreased to around 88%, but was restored upon reintroduction of O_2_. This behavior can be explained by the Mars-Maessen mechanism,^32^ wherein Hg^0^ reacts with lattice oxygen or chemisorbed oxygen to form HgO or weakly bonded mercury complexes. Fig. 3b and c show the effects of SO_2_ and NO on Hg^0^ removal efficiency. It was found that the introduction of 150 ppm SO_2_ or NO would not influence the performance of Hg^0^ removal, while when the concentration of SO_2_ or NO increased to 300 ppm, Hg^0^ removal efficiency would slightly decrease. This decline can be attributed to the poisoning of Mn-based catalysts by SO_2_ or NO, leading to the formation of stable sulfate species or nitrate substances that reduce the active sites.^33,34^ These results displayed that BiOI-modified MnO_*x*_-TiO_2_ exhibits an excellent durability in complex flue gas environments.

To further evaluate the stability and reusability of the catalyst, a multi-cycle test was conducted using the BiMnTi-5 sample. In the simulated flue gas atmosphere at 150 °C, the catalyst was continuously operated for four cycles (1 h per cycle), and its Hg^0^ removal efficiency remained above 95% (as shown in Fig. 3d), without significant activity decay. These results demonstrated that the catalyst exhibited good structural stability and recyclability.

### 3.2. Characterization analysis

#### 3.2.1. SEM, EDS mapping, and HRTEM analysis

**Fig. 4:**
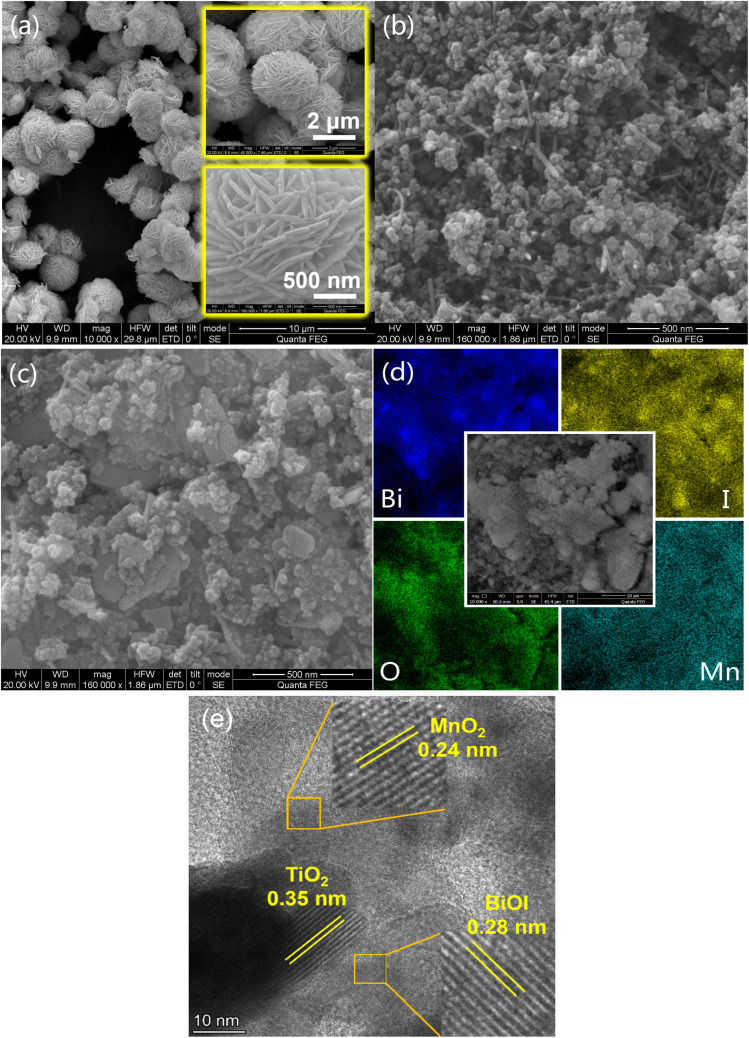
SEM images of BiOI (a), MnTi (b), BiMnTi-5 (c); EDS elemental mapping of BiMnTi-5 (d); HRTEM of BiMnTi-5 (e).

The morphologies and elemental distributions of BiMnTi-5 were characterized by SEM and EDS mapping. As shown in Fig. 4a, BiOI exhibited a spherical architecture assembled from stacked nanosheets. The SEM image of MnTi sample (Fig. 4b) revealed a porous and aggregated morphology composed of two distinct components: quasi-spherical TiO_2_ nanoparticles and MnO_2_ nanorods. The intimate interfacial contact between these two phases effectively suppresses particle agglomeration, thereby enhancing the active surface area and promoting synergistic electronic interactions. As for BiMnTi-5 (Fig. 4c), the MnTi composites were clearly deposited on the surface of sheet-like BiOI. Moreover, the EDS elemental mapping (Fig. 4d) confirmed the uniform distribution of Bi and I elements across the BiMnTi-5 surface, further verifying the successful formation of the composite structure. To further elucidate the microstructural feature of the BiOI-MnO_*x*_-TiO_2_ composite, high-resolution transmission electron microscopy (HRTEM) analysis was conducted. As shown in Fig. 4e, the HRTEM image clearly revealed well-resolved lattice fringes with interplanar spacings of 0.28 nm, 0.24 nm, and 0.35 nm, which can be assigned to BiOI, MnO_2_, and TiO_2_, respectively.^35–37^ Notably, these distinct lattice fringes were observed in close proximity within the same particle, indicating intimate interfacial contact among the three components. This lattice-level evidence confirmed that genuine heterojunctions were formed after the grinding process, rather than simple physical mixing.

#### 3.2.2. XRD and H_2_-TPR analysis

**Fig. 5:**
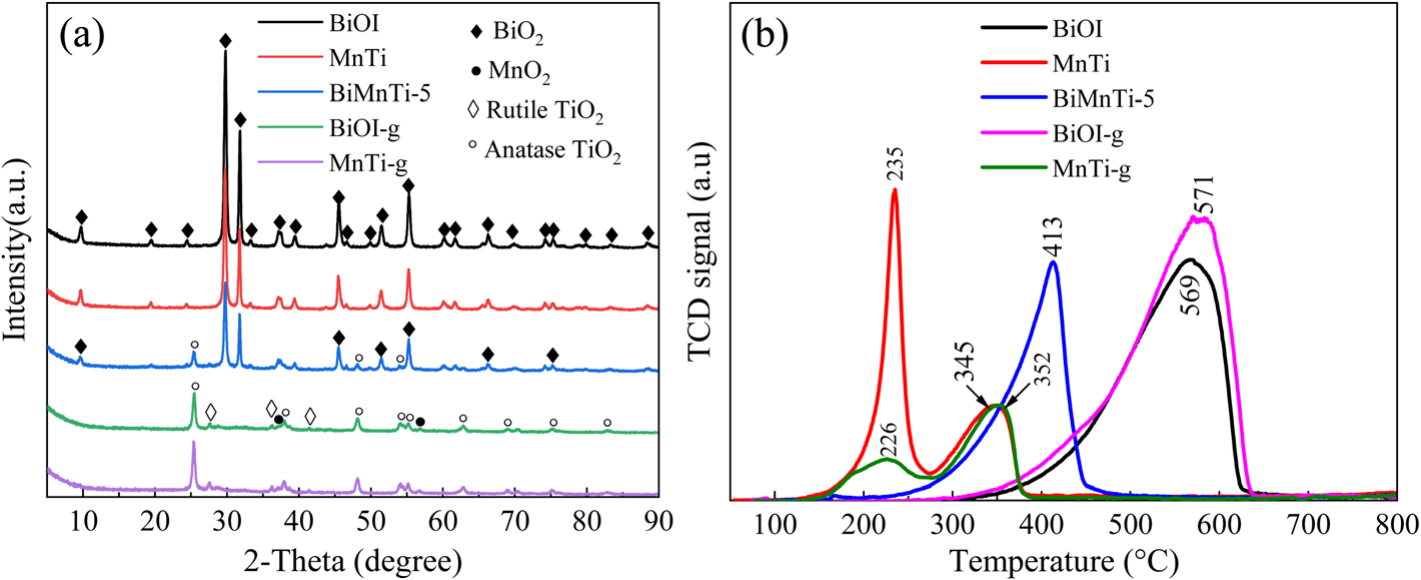
XRD patterns (a) and H_2_-TPR profiles (b) of the catalysts.

Fig. 5a presents the XRD patterns of the catalysts. The characteristic diffraction peaks of BiOI appeared at 2*θ* = 9.7°, 19.5°, 24.4°, 29.7°, 31.7°, 37.1°, 39.4°, 45.4°, 51.4°, and 55.2° (JCPDS No. 10-0445),^22,23^ indicating its high crystallinity. In the MnTi catalyst, the dominant phase corresponded to anatase TiO_2_, while a minor rutile phase was detected at 2*θ* = 25.48° and 27.64°.^31^ Additionally, the diffraction peaks observed at 2*θ* = 37.5 and 56.9° (JCPDS No. 24-0735) can be assigned to MnO_2_, suggesting the partial crystallization of manganese oxide. The XRD patterns of ground BiOI (BiOI-g) and MnTi-g remained consistent with those of pristine BiOI and MnTi, indicating that the grinding process did not alter their crystal structures. Notably, no distinct MnO_2_ peaks are observed in the BiMnTi-5 composite, implying that Mn species were highly dispersed within the material.^31^

Fig. 5b displays the H_2_-TPR profiles of the catalysts. It was found that BiOI showed the highest reduction temperature, suggesting its inherently poor redox activity. For the MnTi catalyst, a sharp reduction peak at 235 °C and a broader one at 345 °C can be assigned to the stepwise reduction of MnO_2_ or Mn_2_O_3_ → Mn_3_O_4_ and the subsequent reduction of Mn_3_O_4_ → MnO, respectively.^38^ The intensity of lower-temperature reduction peak of MnTi-g decreased, showing the change of its redox property during the mechanical grinding process. By comparison, a pronounced reduction peak observed at 413 °C in the H_2_-TPR profile of BiMnTi-5, which was between MnTi and BiOI. Moreover, the initial reduction temperature of BiMnTi started at around 200 °C, much lower than of BiOI. Compared with BiOI, BiMnTi showed a broader peak profile and a larger reduction area, demonstrating that the introduction of MnTi effectively improves oxygen storage and release capability, thereby modulating its redox behavior.^39^

#### 3.2.3. N_2_ adsorption–desorption and FTIR analysis

The physicochemical properties of the catalysts are summarized in Table 1. It can be seen that the BET surface area of BiMnTi-5 was markedly higher than that of BiOI but lower than that of MnTi. Upon BiOI modification, the BiMnTi composite exhibited decreased surface area, pore volume, and pore diameter compared with MnTi. Nevertheless, BiMnTi-5 showed superior catalytic activity and enhanced SO_2_ resistance, confirming that physical adsorption was not the dominant mechanism for Hg^0^ removal. These results highlighted the strong synergistic interaction between BiOI and MnTi, which effectively promoted the overall catalytic performance.

**Table 1:** Pore structure properties of the catalysts^a^

Catalysts	*S*_BET_ (m^2^ g^−1^)	*V*_p_ (cm^3^ g^−1^)	*D*_p_ (nm)
BiOI	13.181	0.067	2.516
MnTi	59.029	0.426	2.51
BiMnTi-5	42.798	0.295	1.191

a *S*_BET_: BET surface area; *V*_p_: pore volume; *D*_p_: average pore diameter.

**Fig. 6:**
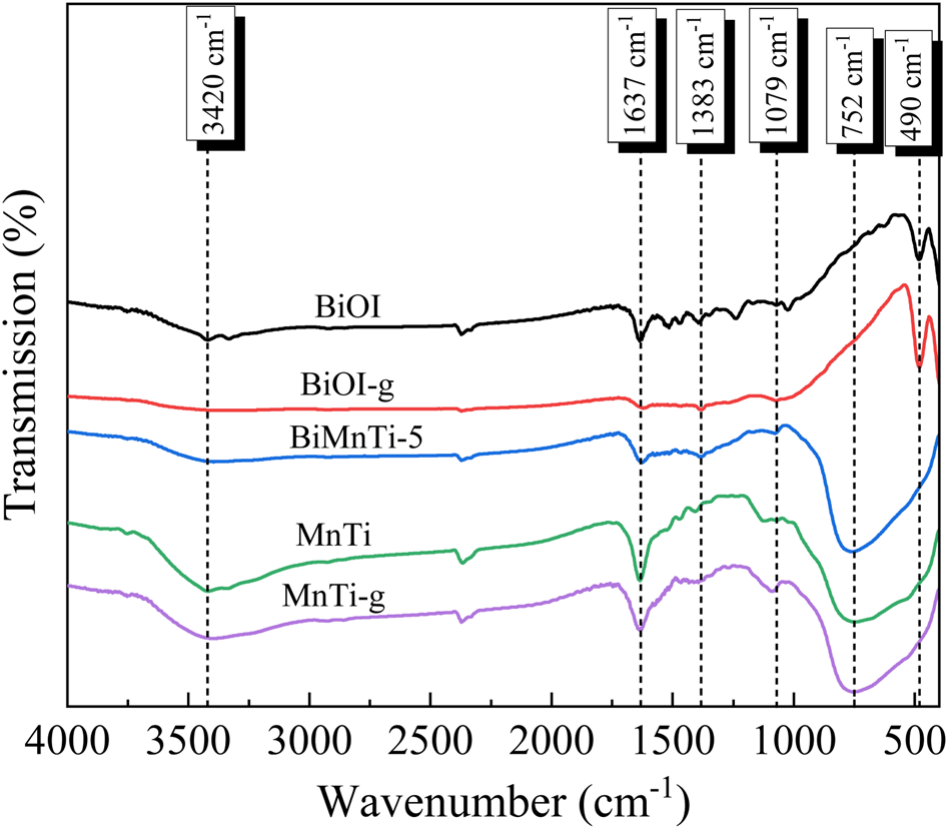
FTIR spectra of the catalysts.

The FTIR spectra of the samples are presented in Fig. 6. The broad absorption band centered at approximately 3420 cm^−1^ corresponded to the stretching vibrations of surface –OH groups. The bands at 1637, 1383, and 1079 cm^−1^ were attributed to the bending vibrations of –OH groups associated with Mn species.^40^ The peak observed at 752 cm^−1^ was attributed to Mn–O–Mn vibrations of MnO_2_, and BiMnTi exhibited an intensive band at 752 cm^−1^, indicating the appearance of MnTi in the composite. The characteristic band at 490 cm^−1^ corresponding to Bi–O stretching disappeared in the BiMnTi-5 sample, implying that the formation of stronger Mn–O covalent bonds may disrupt the original Bi–O bonds.

#### 3.2.4. XPS and EPR analysis

**Fig. 7:**
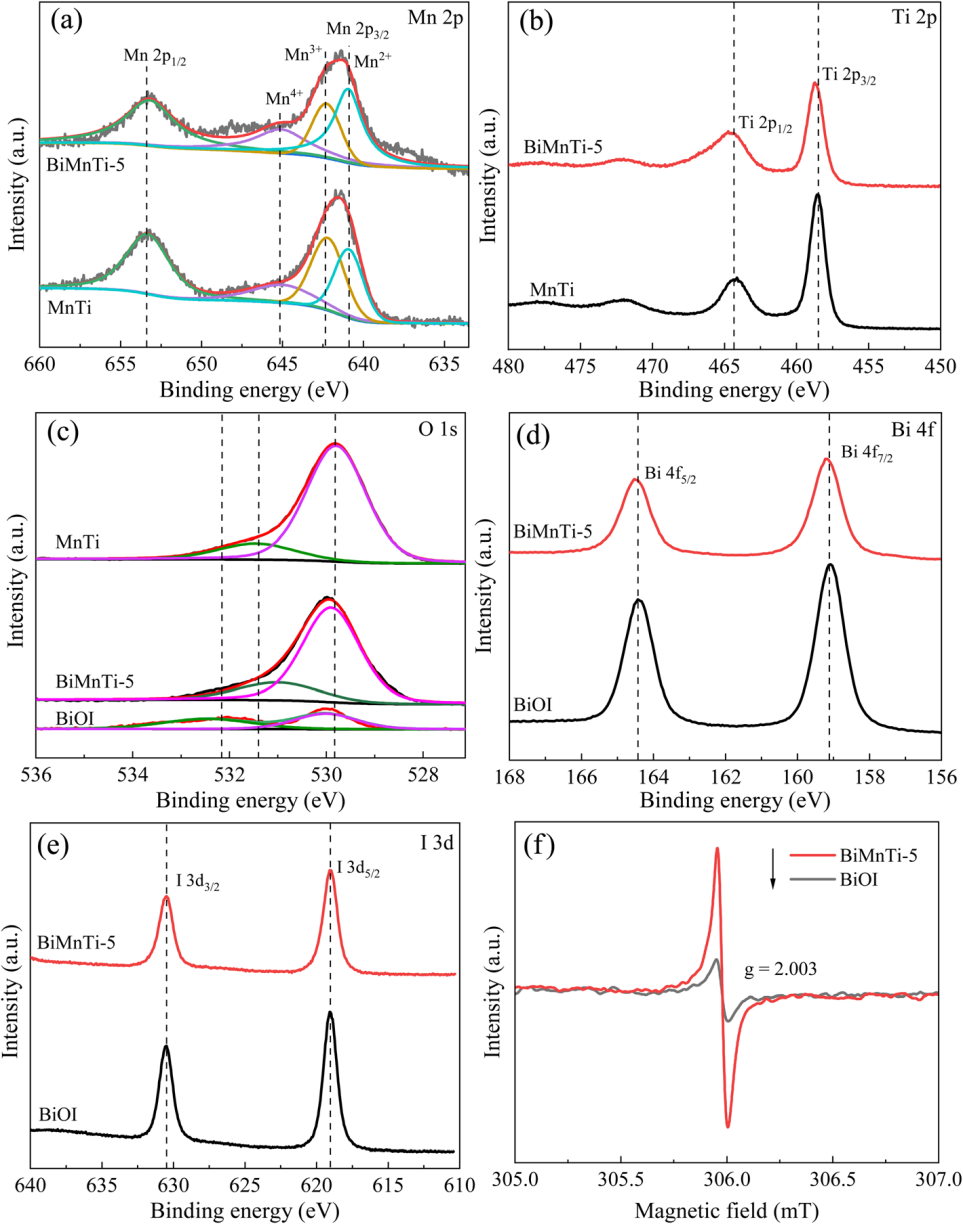
XPS spectra of the catalysts: Mn 2p (a), Ti 2p (b), O 1s (c), Bi 4f (d), I 3d (e). EPR spectra of BiOI and BiMnTi-5 (f).

The XPS spectra of Mn 2p, Ti 2p, O 1s, Bi 4f, and I 3d for BiOI, MnTi, and BiMnTi-5 are shown in Fig. 7a–e. As illustrated in Fig. 7a, the Mn 2p spectrum exhibited two spin–orbit doublets with Mn 2p_3/2_ and Mn 2p_1/2_ peaks located at approximately 641.7 eV at 653.3 eV. The Ti 2p spectrum (Fig. 7b) showed a strong Ti 2p_3/2_ peak at 458.5 eV and a weaker Ti 2p_1/2_ peak at 464.1 eV, corresponding to Ti^3+^ and Ti^4+^, respectively, with Ti^4+^ being dominant. A slight positive shift in the binding energies of Ti 2p_1/2_ and Ti 2p_3/2_ in BiMnTi suggested a decreased electron density around Ti atoms, indicating strong electron transfer between Ti and BiOI.

As shown in Fig. 7c, the O 1s spectrum can be deconvoluted into two components: lattice oxygen (O_α_) at approximately 529.8 eV and chemisorbed oxygen (O_*β*_) at around 531.3 eV.^41^ The O_β_ species are frequently considered the most active oxygen species in oxidation reactions,^31^ and their relative intensity was markedly higher in BiMnTi-5 compared to MnTi. This result indicated that BiOI modification effectively enhanced the oxidative capacity of the catalyst. Notably, although pure BiOI also exhibited a high proportion of chemisorbed oxygen, its Hg^0^ removal performance remained low, likely due to the poor stability or limited accessibility of the active oxygen species. Further deconvolution of high-resolution Mn 2p and O 1s spectra was performed to calculate the relative atomic ratios of Mn^4+^/Mn and O_β_/O based on the integrated peak areas after Shirley background subtraction and sensitivity-factor correction. The obtained ratios for Mn^4+^/Mn and O_β_/O in BiMnTi-5 were 0.28 and 0.24, respectively, which were clearly higher than those of MnTi (0.23 and 0.19). It was clear that the introduction of BiOI significantly modulated the surface electronic structure of the composite material. The increases in the Mn^4+^/Mn and O_β_/O reflected a strong interaction between BiOI and MnO_*x*_.

In Fig. 7d, the Bi 4f spectrum showed two characteristic peaks at 164.4 eV (Bi 4f_5/2_) and 159.1 eV (Bi 4f_7/2_), consistent with Bi^3+^ species.^24^ The peaks of BiMnTi-5 exhibited a slight positive shift relative to pure BiOI, aligning with the FTIR results and further supporting strong electronic coupling between BiOI and MnTi. As shown in Fig. 7e, two distinct peaks at 630.5 eV and 619.0 eV correspond to I 3d_3/2_ and I 3d_5/2_, respectively, confirming the presence of I^−^ ions.^42^

To confirm the presence of oxygen vacancy, electron paramagnetic resonance (EPR) tests were performed.^43,44^ As shown in Fig. 7f, both samples exhibited a signal peak at *g* = 2.003 in the EPR spectra, which was attributed to oxygen vacancy-related paramagnetic defect centers.^45^ The signal intensity of the ternary composite catalyst BiMnTi-5 was significantly higher than that of the single BiOI, indicating that it had the highest oxygen vacancy concentration. This result corroborated the increased proportion of surface chemisorbed oxygen (O_β_) observed in the XPS O 1s spectra and the changing trend of the reduction peak in H_2_-TPR, directly confirming that the introduction of BiOI promoted the formation of oxygen vacancy. The above characterization analysis revealed that the simple mechanical grinding can alter the surface redox properties of MnTi, but it did not enhance its intrinsic Hg^0^ removal activity. The significant performance enhancement could stem from the compact BiOI-MnTi heterostructure formed after grinding. Therefore, the essence of the synergistic effect would be interface-induced electronic coupling and defect synergy, rather than a simple superposition of component properties.

### 3.3. Mechanism analysis

**Fig. 8:**
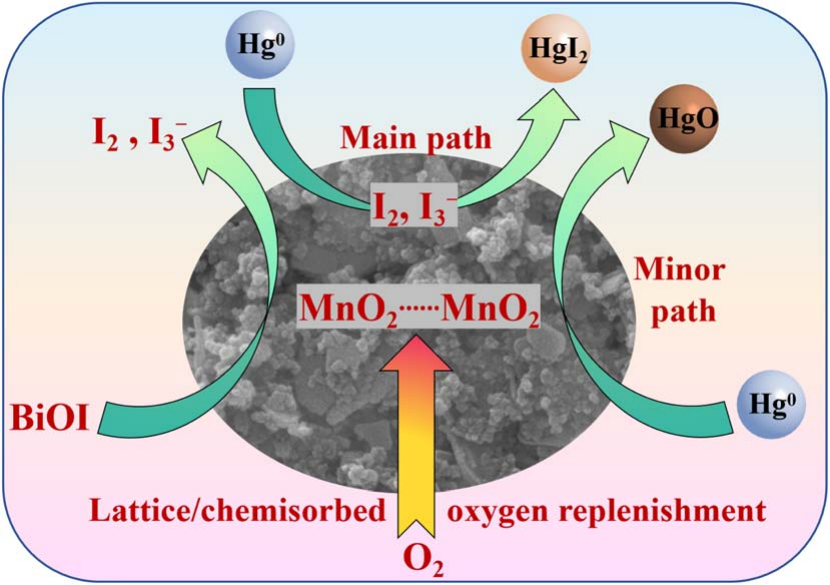
Proposed mechanism for Hg^0^ removal by the BiOI-MnO_*x*_-TiO_2_ catalyst.

A possible mechanism for Hg^0^ removal over the BiOI-MnO_*x*_-TiO_2_ catalyst is illustrated in Fig. 8. Unlike conventional Mn-based catalysts, in which Hg^0^ oxidation mainly proceeds *via* lattice oxygen or chemisorbed oxygen species,^46,47^ the present system involves an iodine-mediated pathway operating under dark conditions. Through close interfacial contact between BiOI and MnO_*x*_, electron transfer enables Mn^4+^ to oxidize I^−^ into active iodine species even in the absence of light. During grinding, BiOI and MnO_2_ undergo strong interactions at the interface rather than simple physical mixing. The I^−^ (iodide ions) in BiOI possess strong reducibility, while the Mn^4+^ in MnO_2_ is a strong oxidant. Thus, electron transfer would occur at their interfaces, most likely leading to the oxidation of I^−^ by Mn^4+^, generating some active iodine species, such as I_2_ (iodine), I_3_^−^ (triiodide ions), or active iodine at defect sites. Moreover, the grinding process itself would introduce some lattice defects, oxygen vacancies, and unsaturated bonds on the surfaces of both materials as observed in the O 1 s spectrum, which could facilitate the adsorption and activation of oxygen molecules, generating surface-active oxygen species that may also participate in auxiliary oxidation processes. The unique layered structure of BiOI provides abundant anchoring sites for Hg^0^ and the generated active iodine species. BiOI could be act as an iodine reservoir, continuously supplying I^−^ for the interfacial reaction with the assistance of MnO_2_. In the process of Hg^0^ removal, although MnO_2_ may also directly oxidize a portion of Hg^0^ to HgO, this is likely not the primary pathway compared to the iodine route. As for BiMnTi catalyst, the interface reaction is of crucial importance. Hg^0^ exhibits an extremely high chemical affinity toward iodine (I_2_) and other active iodine species. This enables a spontaneous and rapid reaction to form HgI_2_, which is the main mercury removal pathway. As a stable solid at the reaction temperature, HgI_2_ is firmly immobilized on the catalyst surface. This reaction proceeds much more readily and with faster kinetics compared to the direct reaction between Hg^0^ and the lattice oxygen of MnO_2_ (minor path). Meanwhile, the gas-phase O_2_ would replenish the lattice or chemisorbed oxygen as found in Fig. 3a. In conclusion, the composite material formed after grinding establishes a synergistic system with distinct functions. BiOI acts as the adsorption center and the iodine source, while MnO_2_ assumes the role of the oxidation center. The relevant chemical reactions were given in eqn (2)–(7). This approach maximizes the oxidative capability of MnO_2_ and the adsorption/iodine storage capacity of BiOI, resulting in a synergistic effect where the combined performance is greater than the sum of its parts.

2 2I^−^ + 2Mn^4+^ → I_2_ + 2Mn^3+^

3 3I^−^ + 2Mn^4+^ → I_3_^−^ + 2Mn^3+^

4 O_2_(g) + * → *O_2_^−^ → *O^−^/*O^2−^

5 Hg^0^ + I_2_ → HgI_2_(s)

6 Hg^0^ + I_3_^−^ → HgI_2_(s) + I^−^

7 Hg^0^ + 2MnO_2_ → HgO + Mn_2_O_3_

## 4. Conclusion

In summary, we successfully fabricated a series of BiOI-modified MnO_*x*_-TiO_2_ composites *via* a facile wet grinding method and evaluated their performance in Hg^0^ removal under simulated flue gas conditions. The BiMnTi-5 composite (with a BiOI to MnTi mass ratio of 5 : 5) exhibited the highest Hg^0^ removal efficiency across a broad temperature window of 50–200 °C, demonstrating remarkable stability and sulfur resistance in the presence of SO_2_ and NO. Characterization results confirmed that the grinding process induced strong interfacial interactions between BiOI and MnTi, leading to enhanced redox properties, increased chemisorbed oxygen species, and improved dispersion of active components. The proposed mechanism highlights the crucial role of interfacial electron transfer, where Mn^4+^ oxidizes I^−^ from BiOI to form active iodine species (*e.g.*, I_2_ or I_3_^−^), which rapidly react with adsorbed Hg^0^ to form stable HgI_2_. Meanwhile, the layered structure of BiOI acts as an effective Hg^0^ adsorbent and iodine reservoir, while MnO_*x*_-TiO_2_ serves as an oxidation center. This synergistic coupling between adsorption and oxidation offers an efficient pathway for Hg^0^ capture without reliance on light irradiation. The findings of this study provide valuable insights into the design of high-performance, non-photocatalytic mercury removal materials with strong practical potential for industrial flue gas treatment.

## Conflicts of interest

There are no conflicts to declare.

## Data Availability

The datasets generated during this study are fully available within the article.
